# Overexpression of miR-210 and its significance in ischemic tissue damage

**DOI:** 10.1038/s41598-017-09763-4

**Published:** 2017-08-25

**Authors:** G. Zaccagnini, B. Maimone, P. Fuschi, D. Maselli, G. Spinetti, C. Gaetano, F. Martelli

**Affiliations:** 1 Laboratory of Molecular Cardiology, Policlinico San Donato-IRCCS, 20097 San Donato Milanese, Milan, Italy; 2 Laboratory of Cardiovascular Research, MultiMedica-IRCCS, 20138, Milan, Italy; 3 Division of Cardiovascular Epigenetics, Department of Cardiology, Internal Medicine Clinic III, Goethe University, Frankfurt am Main, Germany

## Abstract

Hypoxia-induced miR-210 displays a pro-survival, cytoprotective and pro-angiogenic role in several *in vitro* systems. *In vivo*, we previously found that miR-210 inhibition increases ischemic damage. Here we describe the generation of a versatile transgenic mouse model allowing the evaluation of miR-210 therapeutic potential in ischemic cardiovascular diseases. We generated a Tet-On miR-210 transgenic mouse strain (TG-210) by targeted transgenesis in the ROSA26 locus. To functionally validate miR-210 transgenic mice, hindlimb ischemia was induced by femoral artery dissection. Blood perfusion was evaluated by power Doppler while tissue damage and inflammation were assessed by histological evaluation. We found that miR-210 levels were rapidly increased in TG-210 mice upon doxycycline administration. miR-210 overexpression was maintained over time and remained within physiological levels in multiple tissues. When hindlimb ischemia was induced, miR-210 overexpression protected from both muscular and vascular ischemic damage, decreased inflammatory cells density and allowed to maintain a better calf perfusion. In conclusion, we generated and functionally validated a miR-210 transgenic mouse model. Albeit validated in the context of a specific cardiovascular ischemic disease, miR-210 transgenic mice may also represent a useful model to assess the function of miR-210 in other physio-pathological conditions.

## Introduction

Ischemic diseases are characterized by diminished oxygen concentration in the involved tissues, a condition named hypoxia. Hypoxia induces an articulate program of responses aimed at relieving the effects of decreased oxygen tension and removing irreversibly damaged cells^1^. These responses include the expression of a specific subset of miRNAs, named hypoxia-induced miRNAs (hypoxamiRs)^2–4^. HypoxamiRs regulate cell response to decreased oxygen tension modulating the expression of several target genes ^2^. miR-210 can be considered the master hypoxamiR, since it has been found upregulated by hypoxia in all cells and tissues tested to date, carrying out a variety of functions^3–7^. Indeed, miR-210 is a target of Hypoxia-inducible factor 1-α (HIF1α), which directly activates its transcription under low oxygen tension^5^. miR-210, in turn, by targeting several transcripts involved in multiple aspects of cellular response to hypoxia, can inhibit apoptosis^8, 9^, support stem-cell survival^10^, repress mitochondrial metabolism promoting the shift from mitochondria respiration to glycolysis^11^ and induce angiogenesis^8, 12–14^.

Preclinical and clinical evidence confirms the crucial role of miR-210 in the regulation of cell response to hypoxia. Indeed, miR-210 has been found up-regulated in a variety of ischemic conditions and tissues such as hindlimb ischemia in mice^9^, human arteriosclerosis obliterans and atherosclerotic plaques^15–18^, ischemic wounds^19^, after brain transient focal ischemia in rats^20, 21^, upon myocardial infarction in humans^22^ and mice^14^, in failing hearts of diabetic patients^23^, in solid tumors^6^, and in pre-eclampsia^24^.

In a previous study, we identified a prominent role of miR-210 in a mouse model of hindlimb ischemia. miR-210 was inhibited by LNA-antisense oligonucleotides administration followed by femoral artery dissection. We found that miR-210 blocking increased apoptosis and necrosis of both muscular and vascular tissue in the ischemic limb^9^. Indeed, miR-210 blocking counteracted the physiological decrease of oxidative metabolism observed upon hypoxia, inducing mitochondrial dysfunction. This, in turn, led to increased reactive oxygen species (ROS) production, causing apoptosis and tissue damage.

Given miR-210 pro-survival, cytoprotective and pro-angiogenic role in several ischemic conditions, it has been proposed as a potential therapeutic target^9, 12, 14, 18, 25^. In order to obtain an *in vivo* model allowing the evaluation of miR-210 therapeutic potential in ischemic cardiovascular diseases, we generated a miR-210 transgenic mouse strain in which miR-210 expression is under the transcriptional control of a tet-repressor/tet-operator system^26, 27^. This study aims to characterize and functionally validate our transgenic mouse model for further investigations in the area of ischemic diseases. To this aim, we took advantage of our previous observation that miR-210 blocking increased ischemic damage^9^, and tested whether miR-210 transgenic mice exhibited lower muscle damage upon hindlimb ischemia.

## Results

### Generation of transgenic mice conditionally overexpressing miR-210

**Figure 1:**
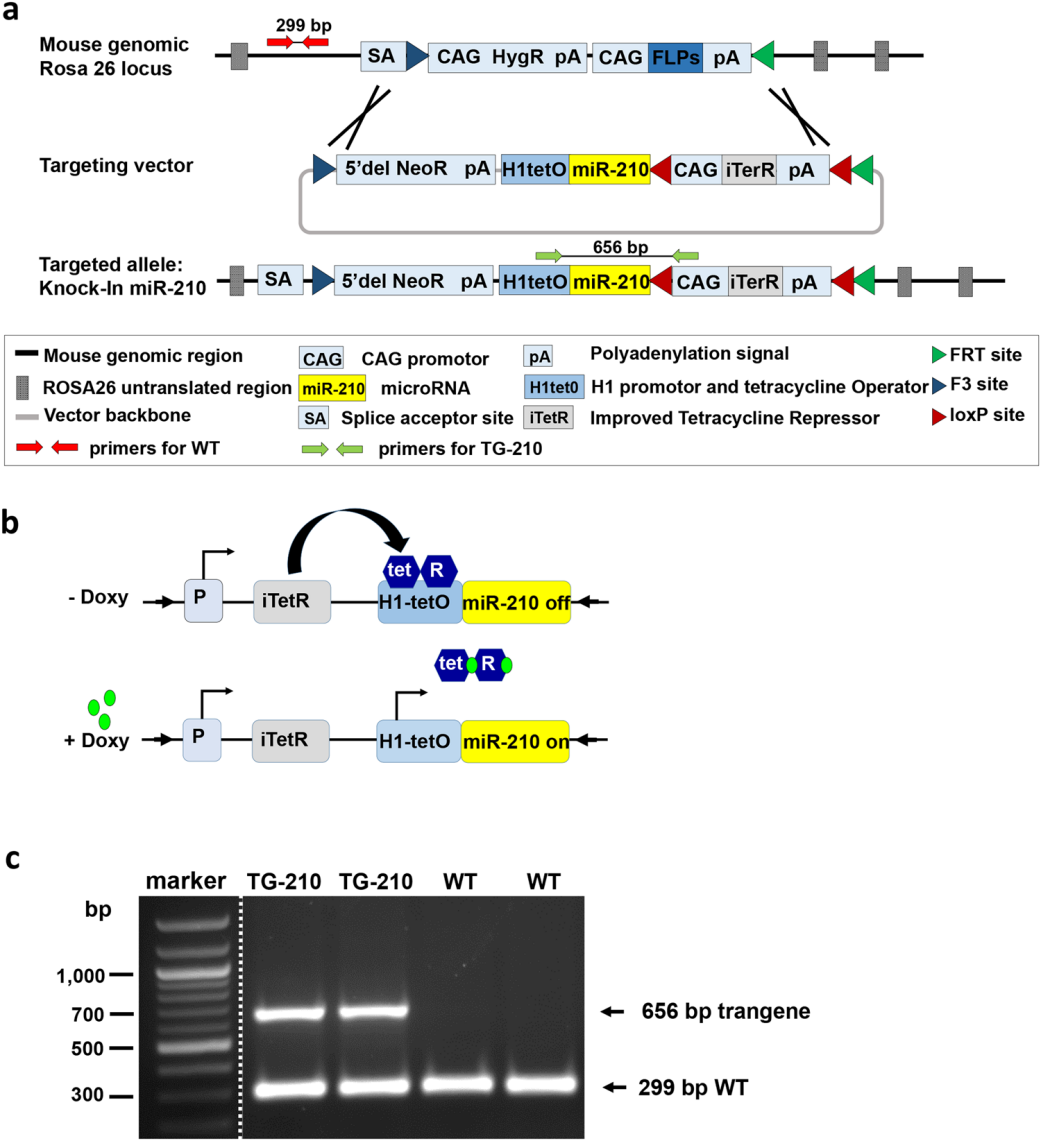
Generation of transgenic mice conditionally overexpressing miR-210. (**a**) The miR-210 coding region, flanked by 110 base pairs of its genomic sequence on each side, was inserted into the ROSA26 mouse locus using a RMCE targeting vector containing the indicated elements. The cassette containing a CAG promoter and an Improved Tetracycline Repressor (iTetR) can be excised by CRE mediated recombination of the loxP sites (red triangles), yielding constitutive overexperession of miR-210. (**b**) Schematic representation of miR-210 regulation in TG-210 mice. In the absence of the doxycycline inducer, the tet-repressor protein binds to the tet-operator control element and inhibits miR-210 expression from the H1 promoter. When doxycycline is administered, it binds to the tet-repressor protein and blocks its interaction with the H1 promoter, allowing miR-210 expression. (**c**) Genotyping strategy. Transgenic mice were genotyped by PCR using the primers indicated in panel a (red primers for WT and green primers for TG-210). The representative gel shows a 299 base pair WT band in all mice and a 656 base pair band present in heterozygous TG-210 mice. Excised lanes are indicated by dashes. Full-length gel is presented in Supplementary Figure 1.

To generate a conditional miR-210 transgenic mouse strain (TG-210), mouse miR-210 was inserted into the ROSA26 locus, by using a targeting strategy that allowed the overexpression of the miRNA either conditionally, upon administration of an inducer (doxycycline), or constitutively, upon recombination of the loxP sites. Figure 1a shows that miR-210 gene was under the control of a promoter containing the tetracycline Operator (H1tetO), followed by a cassette containing an Improved Tetracycline Repressor (iTetR) controlled by CAG promoter. In the absence of doxycycline, the Tet-repressor protein binds to the tet-operator control element, inhibiting the miRNA expression from the H1 promoter (Fig. 1b). When doxycycline is administered, it binds to the tet-repressor protein and blocks its interaction with the H1 promoter^26, 27^. Alternatively, the whole iTetR cassette can be excised by CRE-mediate recombination of the loxP sites, yielding a constitutive activation. Mouse genotype was assessed by PCR (Fig. 1c and Supplementary Fig. 1).

### Conditional overexpression of miR-210

**Figure 2:**
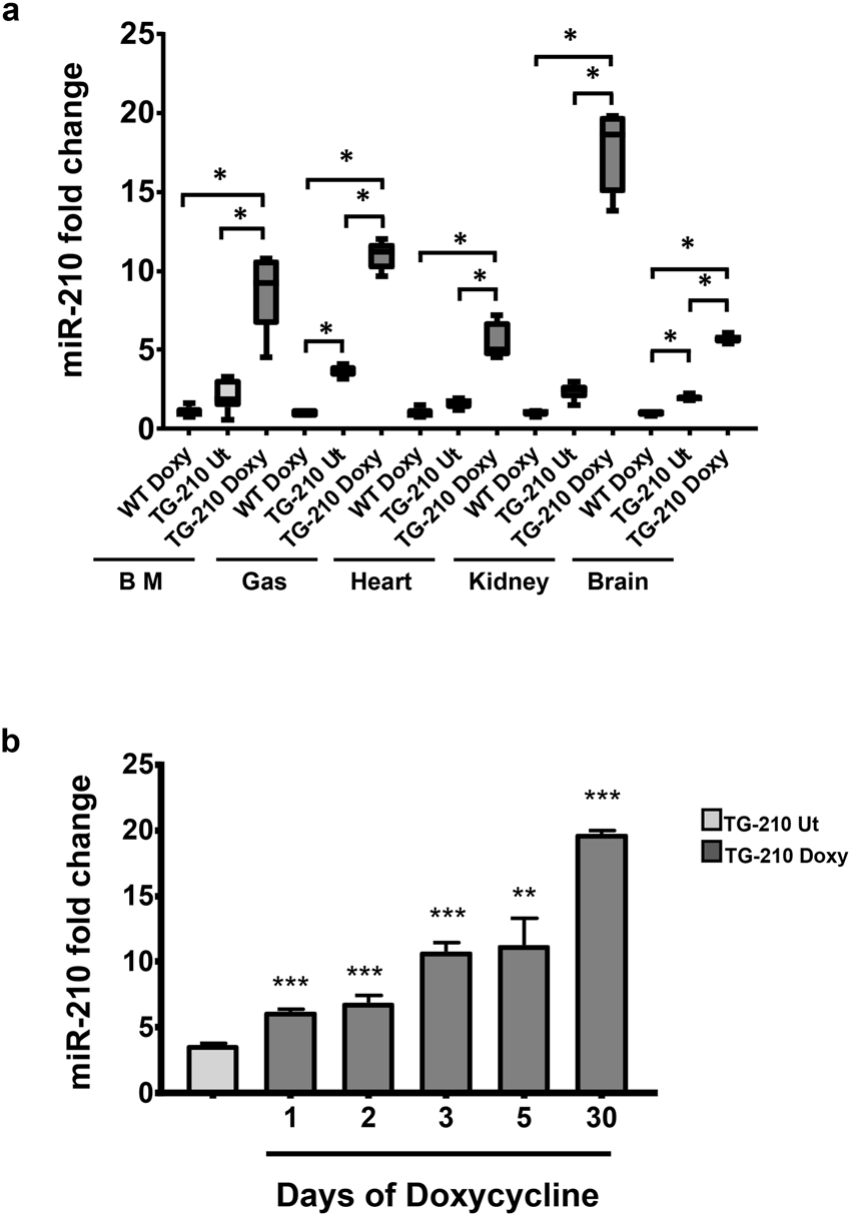
miR-210 induction in TG-210 transgenic mice. (**a**) Ubiquitous increase of miR-210 upon doxycycline treatment. Bone marrow (BM), gastrocnemius muscle (Gas), heart, kidney and brain of WT mice fed with doxycyclin (WT Doxy), TG-210 untreated mice (TG-210 Ut) and TG-210 mice treated with doxycycline-containing food (TG-210 Doxy) for 5 days, were analyzed. The box plot shows miR-210 level measured by qPCR and expressed as fold induction *versus* WT Doxy. Box plots represent data divided in quartiles (n = 5–6; One-way Anova with Tukey’s multiple comparisons *P < 0.0001). (**b**) Time course of miR-210 overexpression in gastrocnemius muscles. Gastrocnemius muscles were harvested from non-ischemic TG-210^Doxy^ mice after the indicated time of doxycycline administration, or from TG-210 UT mice. The bar graph shows miR-210 level measured by qPCR and expressed as fold induction *versus* TG-210 UT (n = 3–5; mean ± SE, Student’s t-test two-tailed ***P < 0.0001; **P < 0.003).

In order to verify whether miR-210 was inducible upon doxycycline administration in our transgenic mouse model, miR-210 expression levels were measured in TG-210 mice treated with doxycycline (TG-210^Doxy^) for 5 days, compared to WT and TG-210 untreated mice (TG-210 UT); RNAs derived from bone marrow, gastrocnemius muscle, heart, kidney and brain were analyzed (Fig. 2a). TG-210 UT mice expressed increased levels of the transgene in absence of doxycycline, displaying a certain degree of leakiness in the model. Nevertheless, when doxycycline was administrated, TG-210^Doxy^ mice showed significantly higher level of miR-210 compared to both WT untreated and TG-210 UT mice in each tissue/organ.

Next, we evaluated miR-210 expression level over time. Figure 2b shows that doxycycline administration led to a rapid and significant induction of miR-210 in the gastrocnemius of TG-210^Doxy^ mice compared to TG-210 UT mice already after one day of treatment. This overexpression was time dependent, since miR-210 levels increased over time. Interestingly, miR-210 induction observed in TG-210^Doxy^ mice was similar to that observed in physio-pathologically relevant conditions^8, 9^.

Taken together, these data indicate that, TG-210^Doxy^ mice conditionally overexpressed miR-210, although some leakiness was observed.

### miR-210 overexpression decreased tissue damage after acute hindlimb ischemia

**Figure 3:**
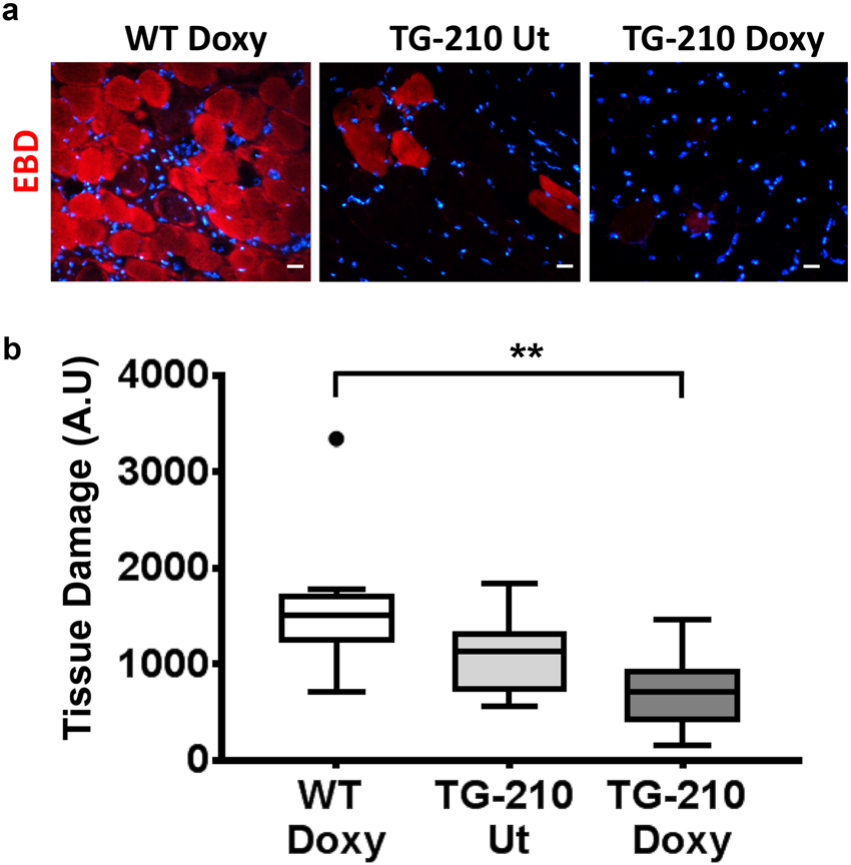
miR-210 overexpression in TG-210^Doxy^ mice decreases tissue damage after ischemia. (**a**) Representative EBD stained sections of ischemic gastrocnemius muscles of WT^Doxy^, TG-210 UT and TG-210^Doxy^ mice, 3 days after ischemia. EBD was administrate intraperitoneally 16 hours before the sacrifice. Red fluorescence indicates damaged myofibers, permeable to EBD; cells with an intact cell membrane remain dark. Nuclei were stained by Hoechst 33342 (blue fluorescence). Magnification 200x, calibration bar 20 µm. (**b**) The box plot shows EBD associated fluorescence expressed as Arbitrary Units (AU) and data are divided in quartiles (n = 9–10; One-way Anova with Tukey’s multiple comparisons **P < 0.0065).

To functionally validate miR-210 transgenic mice, the effect of miR-210 overexpression was evaluated in a mouse model of hindlimb ischemia. To this aim, TG-210 mice were fed with pellets of food containing doxycycline, starting from day 5 before ischemia. Control WT mice were also fed with the same food (WT^Doxy^), to exclude side effects of the drug. Since TG-210 UT mice showed weakly increased levels of miR-210 in each tissue tested, they were also used as control. Hindlimb ischemia was induced by femoral artery dissection in all experimental groups and mice were analyzed at day 3. First, tissue damage was assayed by *in vivo* systemic administration of Evans Blue Dye (EBD)^28^. EBD allows identifying damaged skeletal myofibers that lost cell membrane integrity, becoming permeable to EBD entry. This way, in fluorescence microscopy, damaged tissue emits a red fluorescence, while healthy myofibers remain dark^28^ (Fig. 3a). Figure 3b shows that EBD associated fluorescence was significantly lower in TG-210^Doxy^ gastrocnemius muscles compared to WT^Doxy^ muscles, highlighting less extensive damage. This result suggests that miR-210 overexpression protected skeletal myofibers from ischemic muscle damage. TG-210 UT mice seemed to display damage levels between WT^Doxy^ and TG-210^Doxy^ mice, but differences did not reach statistical significance.

**Figure 4:**
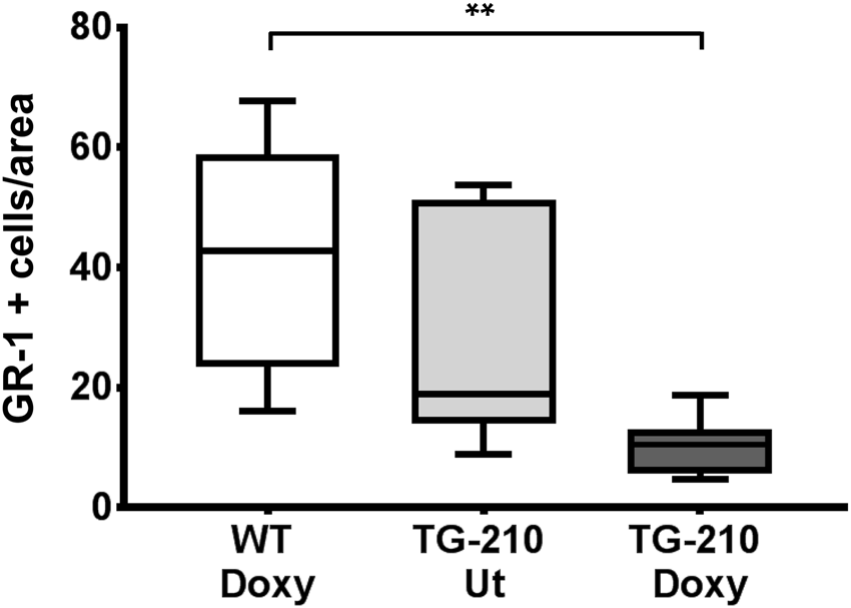
miR-210 overexpression in TG-210^Doxy^ mice decreases neutrophil granulocytes in ischemic tissues. Neutrophil granulocytes density was evaluated by GR-1 immunofluorescence staining of gastrocnemius muscle sections, 3 days after ischemia. The box plot shows GR-1 positive cells/area of WT^Doxy^, TG-210 UT and TG-210^Doxy^ mice. Data are divided in quartiles, (n = 6–7; One-way Anova with Tukey’s multiple comparisons **P < 0.007).

Necrotic cell death stimulates a host inflammatory response involving the recruitment of specific myeloid cell populations^29^. To further confirm the differences observed in tissue damage among the three groups of mice, the presence of neutrophil granulocytes was investigated. Indeed, neutrophils are the first population recruited during the acute inflammatory response^30, 31^. Their density was evaluated in ischemic gastrocnemius muscles by immunofluorescence staining for GR-1. As shown in Fig. 4, significantly lower density of GR-1 positive cells was observed in TG-210^Doxy^ mice compared to WT^Doxy^ controls, according to the lower tissue damage observed. Also in this case, TG-210 UT mice seemed to show an intermediate phenotype.

**Figure 5:**
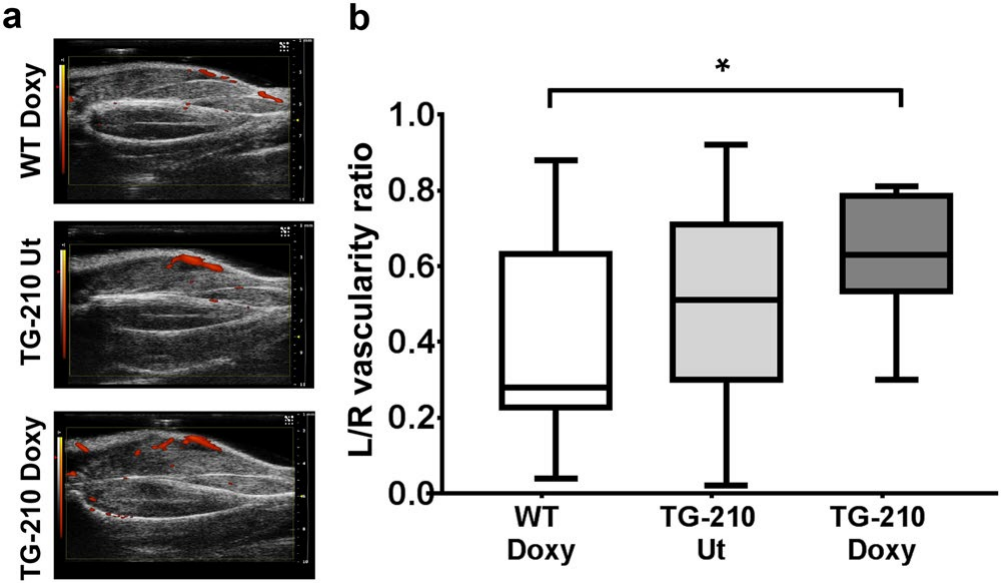
miR-210 overexpression in TG-210^Doxy^ mice increases residual calf perfusion after ischemia. (**a**) Ultrasound perfusion images of mouse calf muscles 3 days after ischemia. (**b**) The box plot shows quantification of vascularity ratio (left ischemic/right non-ischemic), an index of calf perfusion. Box plots represent data divided in quartiles, (n = 10–11; One-way Anova with Tukey’s multiple comparisons **P < 0.04).

To further strengthen these observations, we evaluated vascular damage that follows hindlimb ischemia both by measuring residual calf perfusion and by morphometric analysis in histological sections. Residual calf perfusion was assessed by power Doppler ultrasound (Fig. 5a), measuring the vascularity ratio^32^ (Fig. 5b). The plot shows that residual calf perfusion was significantly higher in TG-210^Doxy^ mice compared to WT^Doxy^ controls, suggesting that miR-210 overexpression allows to maintain a better perfusion in the ischemic limb. Also in this case, TG-210 UT mice seemed to show a phenotype intermediate between WT^Doxy^ and TG-210^Doxy^ mice, suggesting a partial protection, but differences were not statistically significant.

**Figure 6:**
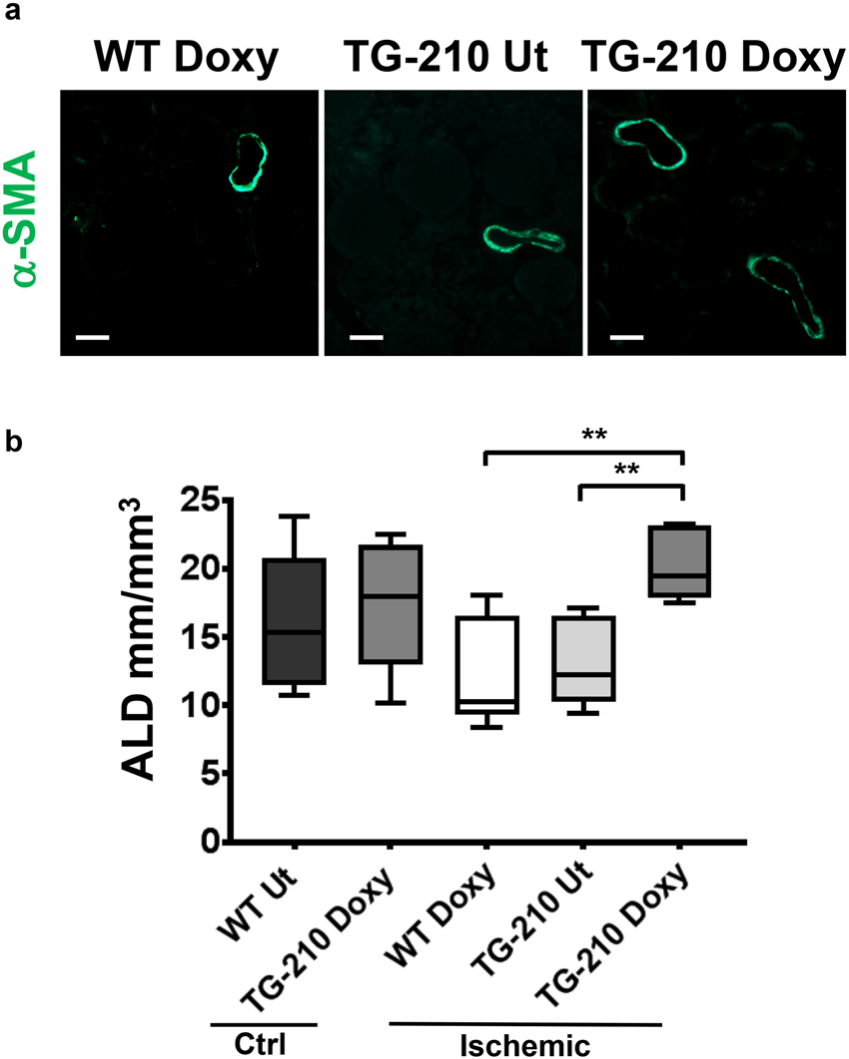
TG-210^Doxy^ mice overexpressing miR-210 maintain a higher arteriolar density after ischemia. (**a**) Representative α-SMA immunofluorescence of WT^Doxy^, TG-210 UT and TG-210^Doxy^ gastrocnemius muscles 3 days after ischemia. Magnification 400x, calibration bar 20 µm. (**b**) The box plot shows ALD quantification and data are divided in quartiles, (n = 5–9; One-way Anova with Tukey’s multiple comparisons *P < 0.05; **P ≤ 0.007).

Next, arteriolar length density (ALD) was quantified in both contralateral non ischemic and ischemic gastrocnemius muscles (Fig. 6). Controlateral non ischemic gastrocnemius muscles of WT and Tg-210^Doxy^ mice showed similar ALD; upon ischemia, TG-210^Doxy^ gastrocnemius muscles conserved a significantly higher ALD compared to both WT^Doxy^ and TG-210 UT mice (Fig. 6b). Of note, ALD differences were in agreement with blood perfusion data (Fig. 5), indicating their functional relevance.

**Figure 7:**
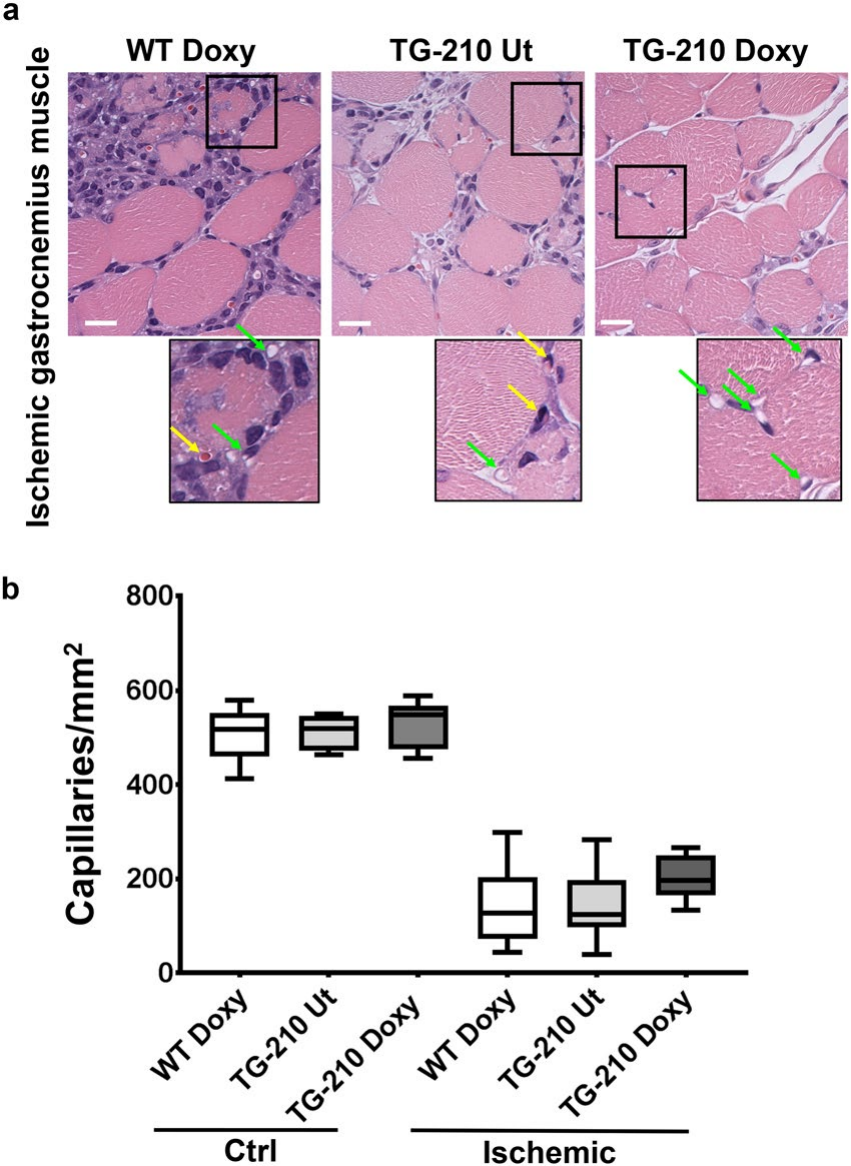
Capillary density after ischemia in miR-210 overexpressing mice. (**a**) Representative Hematoxylin/Eosin stained sections of ischemic gastrocnemius muscles of WT^Doxy^, TG-210 UT and TG-210^Doxy^ mice, 3 days after ischemia. Magnification 400x. Calibration bar 20 µm. Inset shows capillaries at higher magnification. Green arrows indicate capillaries, yellow arrows indicate capillaries with a trapped erythrocyte. (**b**) The box plot shows quantification of capillaries/mm^2^ and data are divided in quartiles, (n = 11).

Capillary density was quantified in hematoxylin/eosin stained sections of ischemic (Fig. 7a) and non-ischemic gastrocnemius muscles. In contralateral non-ischemic muscles, no differences were observed among the three groups of mice. As expected, three days after ischemia capillary density decreased in all experimental group. Nevertheless, TG-210^Doxy^ muscles seem to retain a higher capillary density compared to control groups (Fig. 7b). However, this difference was not statistically significant in comparison with controls, possibly due to insufficient sample numerosity.

Taken together these results suggest that miR-210 overexpression in TG-210^Doxy^ mice protects from ischemic damage, allowing to maintain a better perfusion in the tissue.

## Discussion

In this work, we characterized a novel transgenic mouse model conditionally overexpressing miR-210. To this aim, we took advantage of the TetOn technology that has been successfully used in the past for conditional overexpression of different siRNA and miRNAs^33–35^. We generated this model with the purpose of obtaining a tool by which testing miR-210 therapeutic potential in ischemic cardiovascular diseases. Indeed, we previously demonstrated an anti-apoptotic, pro-survival role of miR-210 both in endothelial cells exposed to hypoxia and in mouse skeletal muscles exposed to acute ischemia^8, 9^. Our findings are also in keeping with several reports indicating that miR-210 inhibition increases apoptosis and cell death in a variety of cell culture systems^4, 5, 36, 37^.

Here, we demonstrated that, administrating an inducer, in this case doxycycline, transgenic mice overexpressed miR-210 efficiently in different tissues and organs. When miR-210 levels were analyzed in gastrocnemius muscles, we observed that they were significantly higher compared to TG-210 UT, although still compatible with physiological upregulation observed under hypoxia^8^ or ischemia^9^. Our analysis also showed that TG-210 uninduced mice expressed miR-210 at levels weakly but significantly higher compared to WT control mice, highlighting a certain degree of leakiness in the system. This problem was previously described by different authors as possibly due to incomplete or limited tetR-mediated suppression of the transgene^33, 38–42^. Thus, to understand if miR-210 could have a cytoprotective role even when weakly increased over basal levels, TG-210 uninduced mice were tested along with the induced ones. We observed that tissue damage, blood flow and inflammation levels were intermediate between those of WT and induced TG-210 mice, suggesting a possible biological function, conceivably via a preconditioning mechanism^10, 25^. However, these trends never reached statistical significance. This indicates that, if present, miR-210 potential preconditioning was not sufficient for a full ischemia protection in the adopted experimental system. More powered experiments with a higher number of samples are necessary to clarify this issue.

For a functional validation of miR-210 transgenic model, we took advantage of the characterization of the effects of miR-210 inhibition in hindlimb ischemia, previously described by our group^9^. For all parameters tested, by increasing miR-210 levels, we obtained results that were inverse to those observed by miR-210 blocking, i.e. lower tissue damage and lower decrease of blood flow and blood vessel density.

Of note, our observations are also in agreement with an *in vivo* study in which a gain of function approach was used after acute myocardial infarction in mice. It was found that miR-210 overexpression, induced by injection of minicircle non-viral vectors in the peri-infarct region, can inhibit apoptosis, increase angiogenesis, and improve cardiac functions^14^.

However, miR-210 function is complex and might be highly context- and time-dependent. In a model of perinatal hypoxic-ischemic encephalopathy, miR-210 blocking by local administration of LNA-anti-miR-210, reduces brain infarct size, improving neurological function recovery^43^. On the other hand, lentiviral brain delivery of miR-210 in mice, before the induction of focal cerebral ischemia, enhances microvessel density, the number of neural progenitor cells and improves neurobehavioral outcomes of the ischemic mouse^44^.

Interestingly, no significant differences were observed in both ALD and capillary density upon miR-210 expression in the non ischemic limbs, indicating no overt induction of angiogenesis in physiological conditions and suggesting a potential therapeutic applicability of miR-210 administration.

To further strengthen our data, the presence of inflammatory cells in the ischemic tissues was also evaluated. Indeed, necrotic cell death stimulates a host inflammatory response that involves the recruitment of specific myeloid cell populations within the injured area^29^. Specifically, neutrophils represent the first inflammatory myeloid cells that invade the area of injury with a peak of expression from 1 day to 5-day post-injury^30, 31^. As expected, TG-210^Doxy^ mice showed significantly lower density of neutrophil granulocytes, according to the decreased tissue damage observed. Specific studies are needed to investigate miR-210 function in the inflammatory response.

Taken together, these results demonstrated that miR-210 overexpression in TG-210^Doxy^ mice protects gastrocnemius muscle from muscular and vascular ischemic damage and allows to maintain a better calf perfusion, identifying miR-210 as potential therapeutic target in ischemic cardiovascular diseases^12, 14, 18, 25^.

Limitations of this study include increased basal miR-210 levels in TG-210 mice, somewhat complicating data analysis. Moreover, the present study should be interpreted as a proof of principle, meant to stimulate further investigations on miR-210 role in ischemia. More studies are also necessary to clarify the mechanisms of miR-210 action, as both direct and indirect mechanisms might underpin miR-210 protective function. miR-210 targeting of oxidative phosphorylation genes, allowing the control of ROS formation upon ischemia, is a likely mechanism; nevertheless, other indirect effects might be at work as well ^2–5, 7, 9, 11^.

Albeit validated in the context of a specific cardiovascular ischemic disease, TG-210 mice may also represent a useful model to assess the function of miR-210 in other physio-pathological conditions, such as cancer^6, 45^ pre-eclampsia^46^ and immune system dysfunctions^15, 47, 48^.

## Material and Methods

### Mouse model

All experimental procedures complied with the Guidelines of the Italian National Institutes of Health and with the *Guide for the Care and Use of Laboratory Animals* (Institute of Laboratory Animal Resources, National Academy of Sciences, Bethesda, Md) and were approved by the institutional Animal Care and Use Committee: Ministero della Salute, Direzione Generale della Sanità Animale e dei Farmaci Veterinari, authorization no. 96/2015-PR (IACUC 666).

Two months old Doxycycline-inducible transgenic C57BL/6NTac-*Gt(ROSA)26Sor* ^*tm3720(Mir210)Tac*^ (TG-210) male mice or C57BL/6N littermate (Wild Type, WT) were used for all experiments. TG-210 mice were generated by Taconic Artemis (Germany). To generate TG-210 mice, the mouse miR-210 coding region flanked by 110 base pair of its genomic sequence on each side, was inserted into the ROSA26 locus using the Recombination-Mediated Cassette Exchange (RMCE) technique ^49^. The targeting vector included (Fig. 1A):

- A promoter and a tetracycline Operator (H1tetO) with a 5xT transcription termination signal, driving miR-210 expression;
- a cassette flanked by loxP sites, containing a CAG promoter, an Improved Tetracycline Repressor (iTetR) and a polyadenylation signal;
- a Neomycin resistance (NeoR) cassette for positive clone selection following successful RMCE.

The targeting vector was co-transfected with the recombinase expressing vector pCAG-Flpe pA into TaconicArtemis C57BL/6 embryonic stem (ES) cell line carrying RMCE docking sites in the ROSA26 locus. Recombinant clones were isolated using positive (NeoR) selection. Correct RMCE events were confirmed by Southern-blot analysis using a standard protocol. Validated ES cells were injected into F1 blastocysts. After recovery, 8 injected blastocysts were transferred to each uterine horn of 2.5 days post-coitum pseudopregnant NMRI females. Chimerism was measured in chimeras (G0) by coat color contribution of ES cells to the BALB/c host (black/white). Highly chimeric mice were bred with C57BL/6 females. Germline transmission was identified by the presence of black, C57BL/6 strain, offspring (G1). Tg-210 heterozygous male mice were bred with two WT females each in order to establish and amplify a colony. Mice were housed in groups of 3–5 mice at 22 ± 2 °C, using a 12 h light-12 h dark cycle. Unless otherwise stated, animals were fed normal chow diet (SDS, irradiate VRF1).

### Genotyping analysis

Genomic DNA was extracted from tail biopsies by using DirectPCR Lysis Reagent (Viagen Biotech) according to the manufacturer’s protocol. PCR reactions were performed to identify the presence of the transgenic miR-210 coding region, using the following primers: forward primer 5′-CCTGCAATATTTGCATGTCG-3′ and reverse primer 5′-GTCCCTATTGGCGTTACTATGG-3′. The unmodified ROSA26 locus was amplified as a control and to determine the zygosity of the locus, using the following primers: forward primer 5′-CTCTTCCCTCGTGATCTGCAACTCC-3′ and reverse primer 5′CATGTCTTTAATCTACCTCGATGG-3′. PCR conditions were as follows: pre-denaturation at 95 °C for 5 min, followed by denaturation at 95 °C for 30 s, primer annealing at 60 °C for 30 s, and extension at 72 °C for 1 min, and finally an additional extension at 72 °C for 10 min.

Reactions were analysed on 1.5% agarose gel containing ethidium bromide.

### Induction of miR-210 transgene overexpression

In order to induce miR-210 overexpression, mice were fed with food pellets containing doxycycline 2 g/kg (Mucedola) ad libitum. Doxycycline was administrated 5 days before all surgical and experimental procedures.

To confirm the efficacy of miR-210 induction in hindlimb ischemia experiments, miR-210 expression was evaluated in non-ischemic contralateral quadriceps femoris muscles of each mouse, at 3 days of ischemia.

### miR-210 quantification

Total RNA was extracted from each tissue using TRIzol (Invitrogen) and the TissueLyser system (Qiagen). miRNA levels were analyzed using TaqMan quantitative real-time PCR (qPCR; 1 ng per assay) and quantified with 7900HT Fast Real Time PCR system (Applied Biosystems).

Primers for miR-210, miR-16, U6 and the reagents for reverse transcriptase and qPCR reactions were all purchased from Applied Biosystems. miR-210 level in each sample was normalized to miR-16 and U6 average expression, as previously described^9, 50^.

### Surgical and perfusion procedures

Before all surgical and perfusion procedures, mice were anesthetized with an intraperitoneal injection of 10 mg/kg Xylazine (Intervet Farmaceutici) and 100 mg/kg Ketamine (Ketavet 100; Intervet Farmaceutici). Dissection of the left femoral artery was previously described^51^.

Perfusion in ischemic calves was measured with Ultrasound device VEVO 2100 (Visual Sonics), using a 2100 transducer in power Doppler mode (transmit Power 100%; center frequency 32 MHz; gate 2; pulse repetition frequency; beam angle 0; Doppler gain 35 dB; dynamic range 35 dB) 3 days after ischemia. Residual calf perfusion was assessed by measuring the vascularity ratio (left ischemic/right non ischemic) as previously described^32^.

For evaluation of myofibers permeability by Evans Blue Dye (EBD), 1% EBD solution (Sigma-Aldrich) in phosphate-buffered saline (PBS, pH 7.5) was prepared, sterilized by Millex-GP 0.22 µm filtration (Millipore) and stored at 4 °C. EBD solution (1% volume relative to body mass) was injected into the right side of the peritoneal cavity 16 hours before sacrifice^28^. Thereafter gastrocnemius muscle samples were harvested and frozen in OCT embedding medium.

For histological analysis, mice were perfused via left ventricle with PBS pH 7.5, followed by 10% buffered formalin, at 100 mm/Hg for 10 min^51^. Next, gastrocnemius muscles were harvested, fixed and paraffin embedded.

### Histology and morphometric analysis

For tissue damage quantification, 10 µm thick frozen sections from EBD samples were cut at –21 °C by a cryostat (Leica, Cryocut 1800), air-dried at room temperature, fixed in cold acetone (–20 °C) for 10 min and washed in PBS. Nuclei were stained by Hoechst 33342 (Sigma-Aldrich) and sections were mounted with fluorescence mounting medium (Dako, S3023)^9^. EBD associated fluorescence was quantified in-10–12 random fields/section at 200x magnification by ImageJ software^52^. The presence of myofibers in areas of the section not stained/dark was identified by both nuclear staining with Hoechst and morphological criteria by phase contrast microscopy. For capillary density quantification, Hematoxylin/Eosin stained sections of paraffin embedded gastrocnemius muscles were prepared as previously described^51^. Capillary density was measured counting the number of capillary profiles in 30–40 random fields/section, at 1000x magnification^9^. α-smooth muscle actin (α-SMA) labeling was used to identify arterioles as previously described ^51^. Briefly, the following antibodies were used: α-SMA antibody (α-SMA clone 1A4; Sigma) and anti-mouse IgG Fab specific FITC conjugate secondary antibody (F5262, Sigma). Arterioles with at least one layer of stained smooth muscle cells were visualized at 400x magnification. Images were acquired on the whole section and arterioles with a minimum internal diameter between 4 and 40.99 μm were considered. Arteriolar length density (LD) was determined by the following formula: LD (mm/mm^3^) = Σ(a/b)/M, where a and b are the maximum and minimum internal arteriolar diameters, respectively, and M is the solid tissue area^53^. For immunofluorescence of neutrophil granulocytes, paraffin embedded gastrocnemius muscles sections were incubated with the following antibodies: rat anti-mouse GR-1 (BD PharmigenTM) diluted 1:50 in PBS containing 5% BSA and goat anti-rat 488 Alexa Fluor diluted 1:100 in PBS.

A Zeiss Axioimager 2 fluorescence microscope equipped with Axiovision image analyzer software was used to acquire images and to measure areas. All histological and morphometric analyses were carried out by two blinded readers with comparable results.

### Statistical analysis

Continuous variables were analyzed by two-tailed Student’s t-test or one-way ANOVA approach with Tukey’s multiple comparisons. All statistical tests were performed 2-sided and a p < 0.05 was considered as statistically significant. Continuous variables were expressed in bar graphs as mean ± standard error (SE) or in box plots representing data divided in quartiles. Outliers were identified by Tukey’s test. GraphPad Prism v.4.03 software (GraphPad Software Inc.) was used for statistical analysis.

## Electronic supplementary material

Supplementary figure 1
