# Supplementary material for: Overexpression of miR-210 and its significance in ischemic tissue damage
Source: Sci Rep. 2017 Aug 25;7:9563. doi: 10.1038/s41598-017-09763-4 (PMC5573334; doi:10.1038/s41598-017-09763-4)
Supplement: Supplementary file 1 — Supplementary figure 1 [file 41598_2017_9763_MOESM1_ESM.pdf]

Title:

Overexpression of miR-210 and its significance in ischemic tissue damage

Zaccagnini G.[1], Maimone B.[1], Fuschi P.[1], Maselli D.[2], Spinetti G.[2], Gaetano C.[3], Martelli F.[1*]

[1] Laboratory of Molecular Cardiology, Policlinico San Donato-IRCCS, 20097 San Donato Milanese, Milan, Italy.

[2] Laboratory of Cardiovascular Research, MultiMedica-IRCCS, 20138 Milan, Italy.

[3] Division of Cardiovascular Epigenetics, Department of Cardiology, Internal Medicine Clinic III, Goethe University, Frankfurt am Main, Germany.

*Correspondence should be addressed to Fabio Martelli (email: fabio.martelli@grupposandonato.it)

Short title: miR-210 transgenic mice are protected from ischemic damage

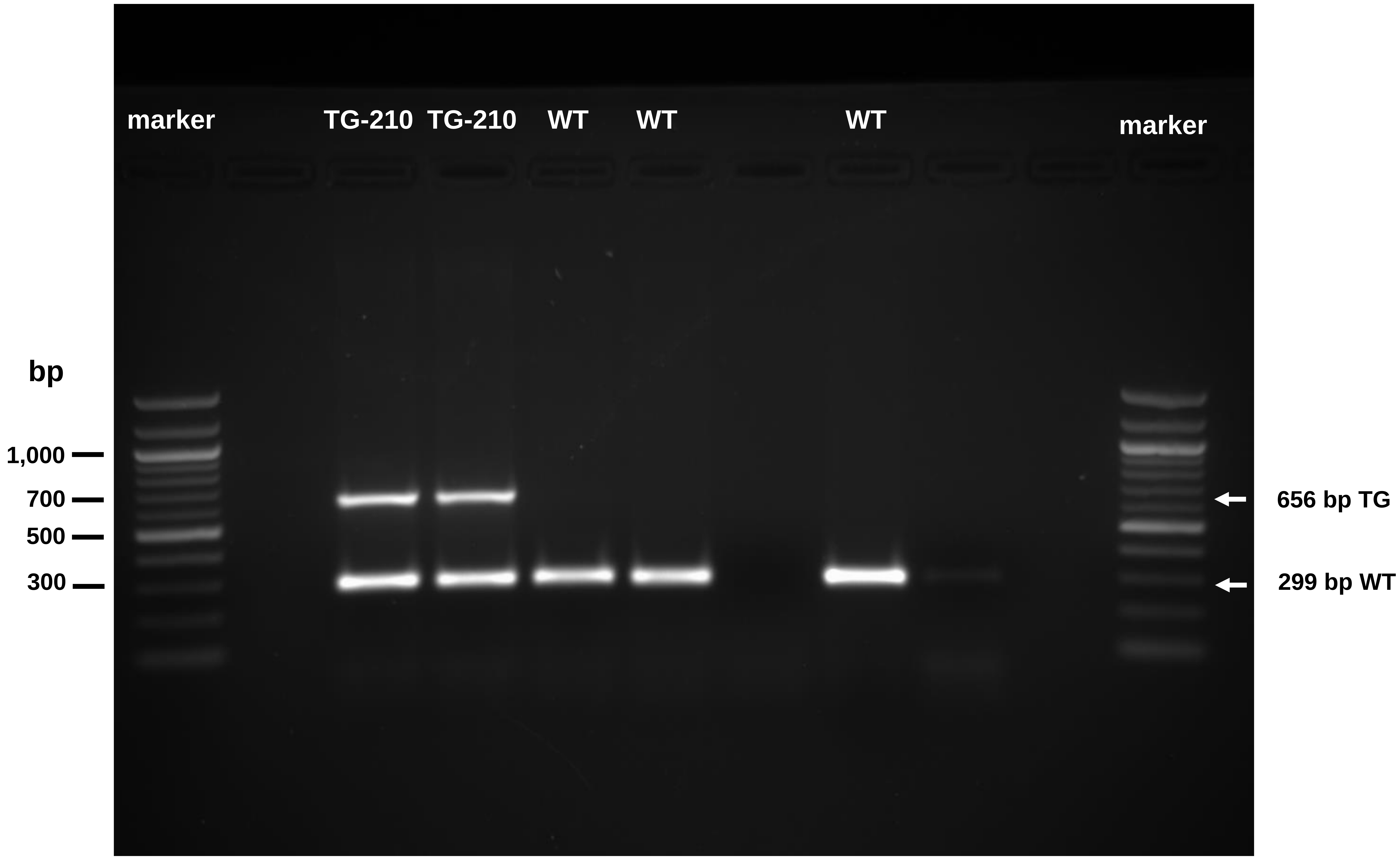

Supplementary figure 1. Full length gel of Fig.1 c. The representative gel shows a 299 base pair WT band in all mice and a 656 base pair band present in heterozygous TG-210 mice.
